# Retraction for Chen et al., “Epsilon-Toxin Production by Clostridium perfringens Type D Strain CN3718 Is Dependent upon the *agr* Operon but Not the VirS/VirR Two-Component Regulatory System”

**DOI:** 10.1128/mbio.00495-22

**Published:** 2022-03-23

**Authors:** Jianming Chen, Julian I. Rood, Bruce A. McClane

**Affiliations:** a Department of Microbiology and Molecular Genetics, University of Pittsburgh School of Medicine, Pittsburgh, Pennsylvania, USA; b Infection Program, Monash Biomedicine Discovery Institute, Department of Microbiology, Monash University, Victoria, Australia

## RETRACTION

Volume 2, no. 6, e00275-11, 2011, https://doi.org/10.1128/mBio.00275-11. In our 2011 *mBio* paper we reported that inactivating the *agrB* gene in the *agr* operon decreases epsilon toxin (ETX) production by Clostridium perfringens type D strain CN3718. After publication, it was noted that some panels in that paper were inadvertently duplicated, so a correction was published to address this problem (see Volume 6, no. 1, https://doi.org/10.1128/mBio.02491-14). However, panels E and F shown in Fig. 5C of the 2014 correction were also recently noted to be inadvertent duplications. In addition, recent findings from our laboratory indicate that our 2011 conclusion, i.e., that the *agr* operon is necessary for CN3718 and, by extension, other type D strains to produce wild-type ETX levels, is not reliable. Specifically, as described in detail in a *Matters Arising* article submitted together with this retraction (I. Mehdizadeh Gohari, J. Li, J. I. Rood, and B. A. McClane, mBio 13:e00496-22, https://doi.org/10.1128/mBio.00496-22), when we recently made a second *agrB* null mutant using a different stock culture of CN3718, the mutant still produced wild-type ETX levels. To confirm that the *agr* operon is not usually necessary for type D strains to produce wild-type ETX levels, we then constructed an *agrB* null mutant in a second type D strain (CN2068) and determined that this mutant also produces wild-type ETX levels. However, the newly-constructed *agrB* mutants of both type D strains produced reduced amounts of alpha toxin and this effect was reversible by complementation, which confirms loss of functional AgrB expression in these mutants since alpha toxin production is known to be regulated by the Agr quorum sensing system. For these reasons we retract the previous paper and apologize for any inconvenience this may have caused readers.

